# Proximal adductor avulsions are rarely isolated but usually involve injury to the PLAC and pectineus: descriptive MRI findings in 145 athletes

**DOI:** 10.1007/s00167-020-06180-5

**Published:** 2020-08-06

**Authors:** Ernest Schilders, Adam W. M. Mitchell, Rowena Johnson, Alexandra Dimitrakopoulou, Christiana Kartsonaki, Justin Charles Lee

**Affiliations:** 1 Department of Orthopaedic Surgery, Fortius Clinic, 17 Fitzhardinge Street, London, W1H 6EQ UK; 2 Department of Radiology, Fortius Clinic, 17 Fitzhardinge street, London, W1H 6EQ UK; 3 Nuffield Orthopaedic Centre, Oxford University Hospitals, Oxford, UK; 4 MRC Population Health Research Unit, Nuffield Department of Population Health, University of Oxford, Oxford, UK; 5 School of Sport, Leeds Beckett University, Leeds, UK

**Keywords:** Adductor longus avulsion, Adductor injuries, Professional athletes, Football, Magnetic resonance imaging, Groin pain, Acute sports injuries, Pyramidalis, PLAC, Sports

## Abstract

**Purpose:**

The purpose of the study is to review the MRI findings in a cohort of athletes who sustained acute traumatic avulsions of the adductor longus fibrocartilaginous entheses, and to investigate related injuries namely the pyramidalis–anterior pubic ligament–adductor longus complex (PLAC). Associated muscle and soft tissue injuries were also assessed.

**Methods:**

The MRIs were reviewed for a partial or complete avulsion of the adductor longus fibrocartilage, as well as continuity or separation of the adductor longus from the pyramidalis. The presence of a concurrent partial pectineus tear was noted. Demographic data were analysed. Linear and logistic regression was used to examine associations between injuries.

**Results:**

The mean age was 32.5 (SD 10.9). The pyramidalis was absent in 3 of 145 patients. 85 of 145 athletes were professional and 52 competed in the football Premier League. 132 had complete avulsions and 13 partial. The adductor longus was in continuity with pyramidalis in 55 athletes, partially separated in seven and completely in 81 athletes. 48 athletes with a PLAC injury had a partial pectineus avulsion. Six types of PLAC injuries patterns were identified. Associated rectus abdominis injuries were rare and only occurred in five patients (3.5%).

**Conclusion:**

The proximal adductor longus forms part of the PLAC and is rarely an isolated injury. The term PLAC injury is more appropriate term. MRI imaging should assess all the anatomical components of the PLAC post-injury, allowing recognition of the different patterns of injury.

**Level of evidence:**

Level III.

## Introduction

Proximal adductor longus avulsions are challenging injuries to diagnose correctly and manage appropriately. Misdiagnosis and delay in treatment can result in significant functional disability in athletes. Adductor injuries are common in football, accounting for up to 23% of all the muscle injuries [6].

An imaging study assessing adductor injuries in football found that 26% of adductor injuries were proximal [25]. Although both conservative [24, 26] and surgical management [1, 5, 10, 15, 16, 27] of proximal adductor avulsions can result in satisfactory outcomes, there is still considerable debate as to which treatment option provides superior outcomes and the fastest return to sport. A better understanding of the anatomy of the symphyseal and perisymphyseal area can help in the correct diagnosis and subsequent appropriate management of these complex injuries.

**Fig. 1:**
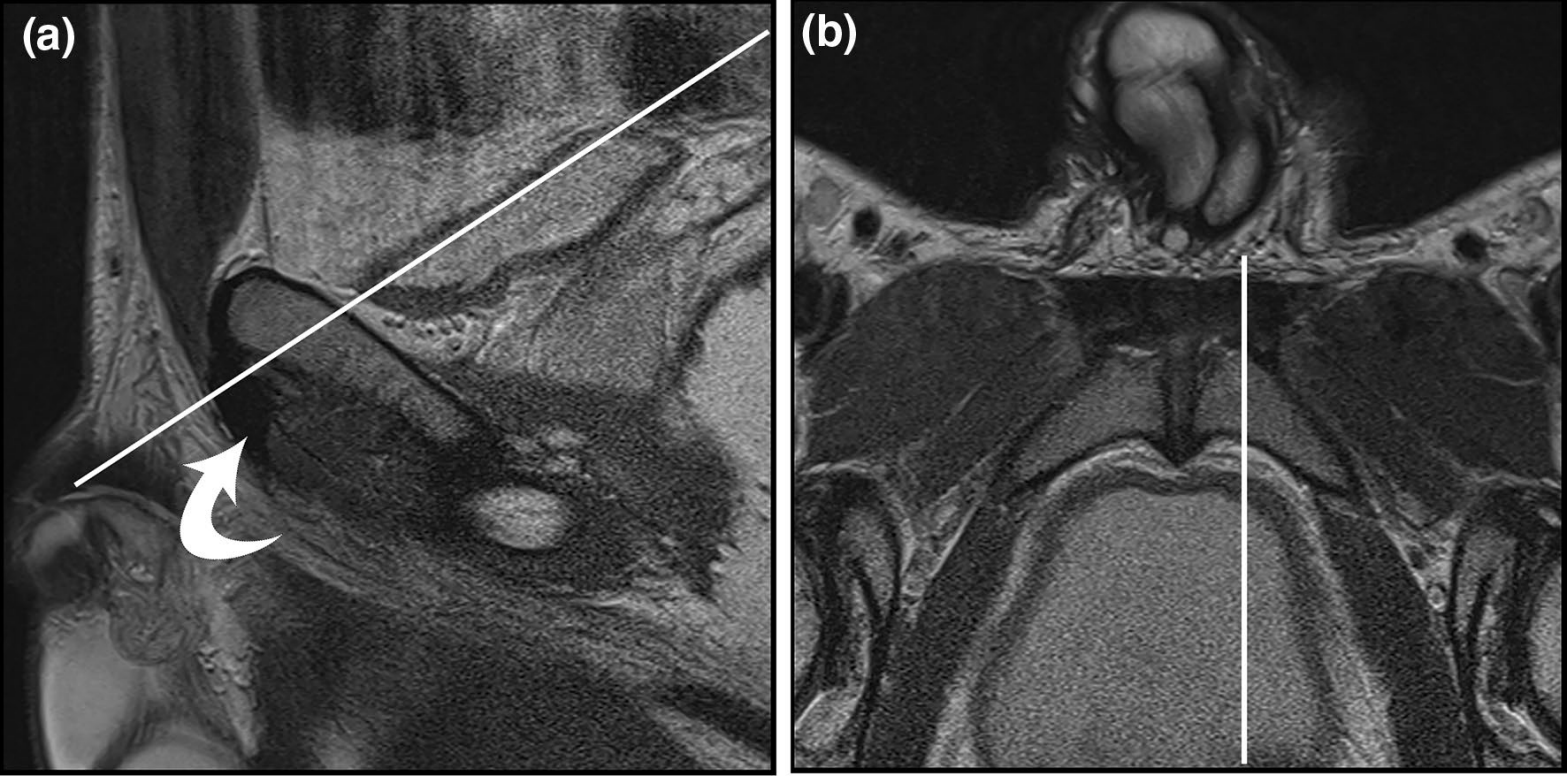
**a** Sagittal and **b** oblique axial T2 MR. Oblique axial image acquired perpendicular to adductor longus fibrocartilage (curved arrow) as indicated by solid white line. Note that the sagittal image is just to the left of midline and, therefore, includes the fibrocartilage

Schilders et al. recently introduced an anatomical concept of the pyramidalis–anterior pubic ligament–adductor longus complex (PLAC) [20]. The pyramidalis is a small triangular muscle, located anterior to the rectus abdominis in the midline and the only abdominal muscle that lies anterior to the pubic bone, which is a key feature for magnetic resonance imaging (MRI) interpretation. Its inferior origin is at the pubic crest and the anterior pubic ligament, and superiorly it attaches to the linea alba. The proximal adductor longus attaches to the anterior pubic ligament and the pubic crest. The adductor longus tendon is in continuity with the anterior pubic ligament. The adductor longus fibrocartilage is anchored to the anterior pubic body, inferior to the pubic crest [3, 5, 19–21] and has a triangular shape or “shark fin” appearance on sagittal views [5, 21, 23]. On the axial oblique views, the fibrocartilage and pubic bone have a butterfly appearance. This classic “shark fin” and butterfly morphology are key features in the recognition of the anatomy on MRI, and aids in the understanding of the imaging patterns in trauma (Fig. 1).

Proximal adductor avulsions can be assessed on ultrasound or MRI [2, 5, 24, 26, 27]; MRI being the preferred choice of imaging. Schilders et al. [20] observed that often in athletes with proximal adductor avulsions, the pyramidalis muscle remained attached to the adductor longus, contradicting the earlier concept that these are isolated adductor longus injuries [25].

We hypothesise that adductor longus injuries are rarely isolated and are usually part of a wider injury to the PLAC. This is an uncommon but important injury that frequently requires repair of multiple structures that form the PLAC. Imaging provides surgical templating allowing planning for which structures need repair.

The purpose of the study was to review the MRI findings in a cohort of athletes who sustained acute traumatic injuries of the adductor longus fibrocartilaginous entheses and to investigate associated injuries to the accompanying structures of the PLAC as well as other muscles, whilst defining the injury characteristics.

## Materials and methods

The study was a clinical audit performed in accordance with the UK Medical Research Council guidelines. Permission to perform the study was given by Fortius clinic and additional ethics approval was granted by Leeds Beckett University: application reference 71730. The MRI scans of all the athletes who presented with an acute injury in our hip and groin clinic between October 2011 and February 2019 were reviewed simultaneously by a musculoskeletal radiologist and an orthopaedic surgeon (first author), both with significant expertise in groin injuries in sports. The studies were blinded and scored by mutual consensus.

The first author assessed all the athletes in the same way utilising a standard clinical assessment protocol for groin pain in athletes—with the use of techniques for groin examination described and validated by Holmich et al. [9].

The clinical presentation was acute adductor-related groin pain [28]. Acute was defined as a specific recent traumatic event such as an overstretching of the leg leading to the injury, after which the athlete was not able to continue training or playing.

On clinical examination, there was typically pain at the adductor longus enthesis as well as pain and weakness on resisted adduction, often with a haematoma in the adductor and lower abdominal area and ecchymosis. MRI scan was performed when there was a clinical suspicion of a traumatic injury to the PLAC. A specific imaging protocol was employed using a modification of the protocol used for chronic adductor enthesopathies [12, 17].

Inclusion criteria were: (1) athletes with a history of acute adductor-related groin pain, (2) traumatic avulsion of the fibrocartilage of the adductor longus on MRI, (3) an MRI scan using a specific protocol which included three mm sagittal and axial oblique sequences.

Exclusion criteria were: (1) adductor-related groin pain with an insidious onset, (2) acute adductor-related groin pain without injury to the adductor longus fibrocartilage, and (3) MRI imaging protocol without appropriate sagittal and/or axial oblique sequences.

### Imaging technique

All MR imaging examinations were performed on a 1.5-T system (Intera, Philips, Best, Netherlands/Avanto, Siemens, Germany). Our standard imaging for acute traumatic adductor longus injuries consists of an axial oblique T1-weighted turbo spin echo (TSE) sequence and an axial oblique T2-weighted or intermediate-weighted TSE fat-suppressed (FS) sequence (Table 1).

**Table 1:** MRI imaging protocol for assessment of the PLAC

Sequence	Plane	Matrix	Slice thickness/gap (mm)	TR	TE
T1-weighted TSE	Axial oblique and sagittal	320 × 320 small	3/0	400	20
T2-weighted fat-suppressed TSE OR	Axial oblique and sagittal	320 × 320 small	3/0	3500	60
Intermediate-weighted fat-suppressed TSE	Axial oblique and sagittal	320 × 320 small	3/0	3500	35
T2-weighted fat-suppressed TSE (optional)	Coronal	512 × 512 large	4/5	3500	60

Three millimetre slices were obtained parallel to the superior aspect of the symphysis pubis and superior rami. Sagittal T1-weighted TSE and sagittal T2-weighted FS sequences were also obtained. The sagittal sequences with three millimetre slice thickness were obtained starting from the symphyseal disc and moving laterally bilaterally. On the sagittal sequences the fibrocartilage has the typical appearance of a shark’s fin (Fig. 1).

### Image analysis

The use of three millimetre slice thickness with no interspace gap optimises signal–noise ratio on a 1.5-Tesla MRI system (Table 1). A large field of view coronal fluid-sensitive sequence with fat suppression can be added to the protocol to screen for other pathology, or as an initial survey.

The axial oblique plane runs perpendicular to the long axis of the fibrocartilage of the adductor longus and gives an optimal visualisation of the pubic symphysis (Fig. 1).

In the setting of acute traumatic injuries, there is often corresponding haematoma formation which is high signal intensity on the fluid-sensitive sequences, facilitating the diagnosis. Intravenous gadolinium is, therefore, unnecessary in diagnosing acute traumatic injuries.

When the fibrocartilage avulses it often displaces laterally, inferiorly and anteriorly. The sagittal images allow a view of the anteroposterior dimensions of the fibrocartilage moving from central to lateral, and are used to infer inferior displacement of the fibrocartilage when present. The axial oblique cuts are made perpendicular to the fibrocartilage–bone interface, demonstrating the anteroposterior and mediolateral dimensions of the fibrocartilage moving from cranial to caudal. They measure the degree of lateral and anterior displacement. The combination of sagittal and axial oblique images is used to assess fibrocartilage displacement in three dimensions.

Previous magnetic resonance studies demonstrated that all components of the PLAC can routinely be visualised [4, 18, 25]. The PLAC was assessed for injury on MRI. The fibrocartilage was interrogated for partial or complete avulsion. The presence or absence of a pyramidalis muscle was confirmed. When present, it was assessed for continuity with the adductor longus, and if torn, the degree of separation was noted. The separation refers to a disruption between the anterior pubic ligament and the adductor tendon. The presence of a concurrent partial injury of the pectineus muscle with associated longitudinal splitting of the inguinal ligament was determined. There were no complete avulsions of the pectineus muscle.

The groups created via these criteria were subsequently assessed for association with disruption of the anterior pubic ligament bridge spanning the pubic symphysis, increased signal in the pyramidalis muscle, rectus abdominis injury and age. Displacement of the adductor longus was defined in 3 categories as none, minimal (< 5 mm) and displaced (> 5 mm).

### Statistical analysis

Data were presented as counts and percentages or as means and standard deviations (SD). The associations of PLAC injury group with symphyseal bridge disruption, increased signal of the pyramidalis, displacement of the adductor longus and rectus abdominis injury were assessed using logistic regression. The association of PLAC injury group with age was assessed using linear regression. Wald tests from regressions were used to assess significance of the associations with each group. Likelihood ratio tests were used to test for heterogeneity between groups.

## Results

One-hundred and forty-five MRI scans fulfilled the inclusion criteria. The average age of the athletes was 32.5 years (18–68, SD 10.9). The pyramidalis was absent in three of 145 patients. Eighty-five athletes (58.6%) were professional, 52 (35.8%) of these athletes competed in the premier league or equivalent league in different countries, 22 (15.1%) competed at international level. All athletes were male.

There were 132 athletes (91.0%) with a complete adductor longus fibrocartilage avulsion and 13 (9.0%) with a partial avulsion. In 55 athletes, the pyramidalis was still connected to the adductor longus; in seven, there was a partial separation; in 81 athletes, the pyramidalis was disconnected from the adductor longus. 48 athletes with PLAC injuries had an associated partial avulsion of the pectineus and inguinal ligament injury. Table 2 shows the different subtypes of PLAC injuries.

**Table 2:** The six different types of PLAC injury according to MRI findings

Type 1	Complete fibrocartilage (FC) avulsion–Pyramidalis separated from Adductor Longus–intact Pectineus
Type 2	Complete FC avulsion–Pyramidalis separated from Adductor Longus–partial Pectineus tear
Type 3	Complete FC avulsion–Pyramidalis connected to Adductor Longus–intact Pectineus
Type 4	Complete FC avulsion–Pyramidalis connected to Adductor Longus–partial Pectineus tear
Type 5	Complete FC avulsion–Pyramidalis partially separated from Adductor Longus–partial Pectineus tear
Type 6	Partial FC avulsion–Pyramidalis connected to Adductor Longus–intact Pectineus

The differentiators are based on the status of the adductor longus fibrocartilage (FC), the pyramidalis and pectineus muscles and tendons

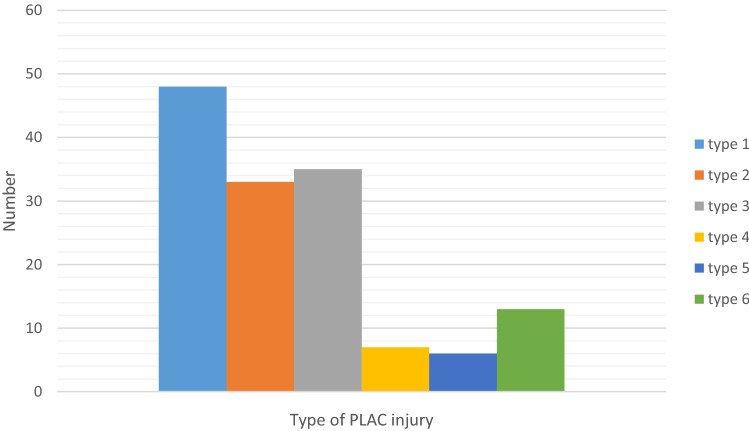

**Fig. 2:**
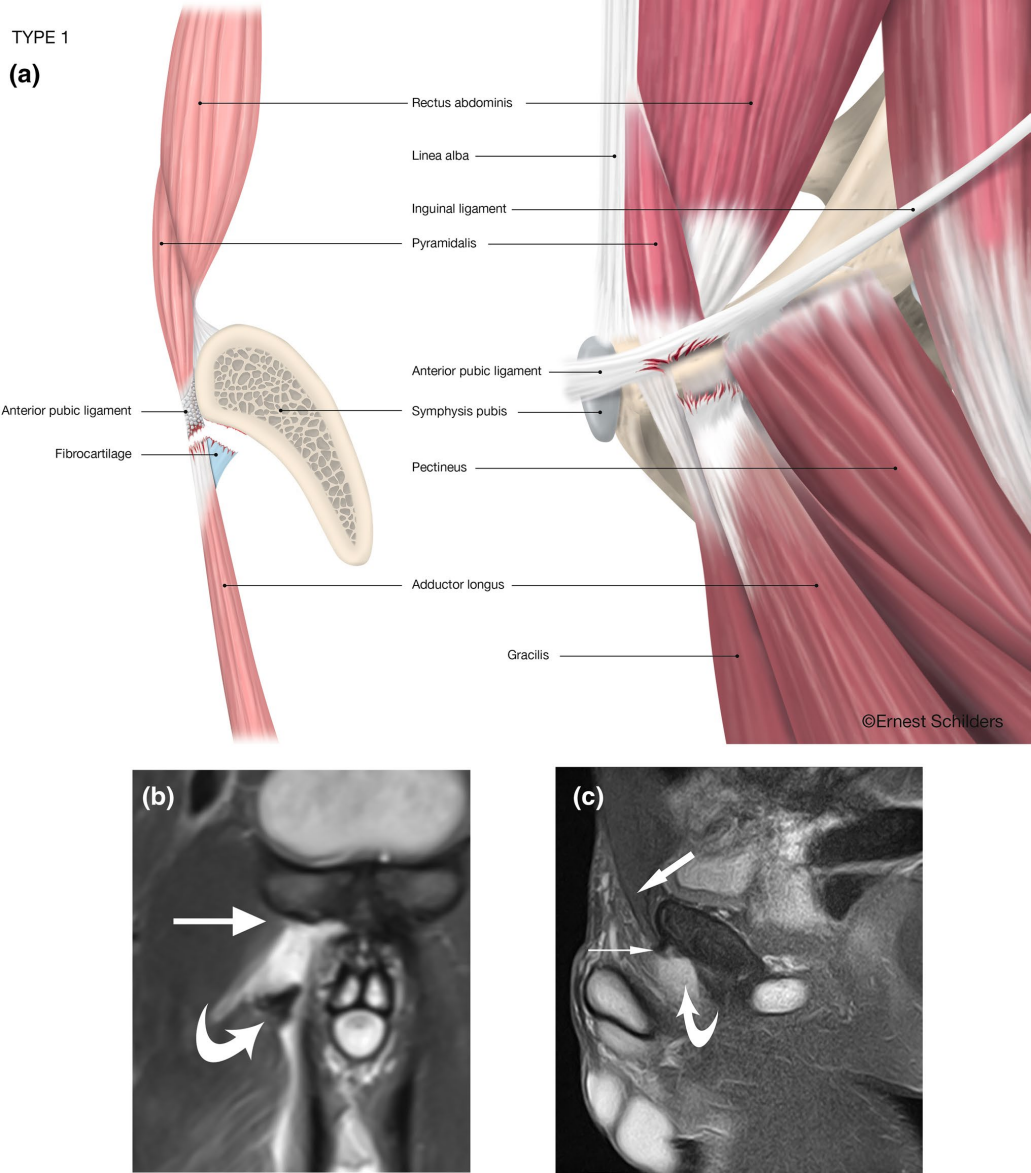
Type 1 PLAC injury: Complete fibrocartilage (FC) avulsion–Pyramidalis separated from Adductor Longus–intact Pectineus. **a** Anatomical line drawing. **b** Coronal fat suppressed proton density MRI. The right adductor longus fibrocartilage has been avulsed and retracted (curved arrow). The pectineus muscle is normal (straight arrow). **c** Sagittal FSPD MRI. Pyramidalis is intact (broad arrow). The anterior pubic ligament origin is intact (small arrow). The adductor longus fibrocartilage is missing due to retraction and lateral displacement (curved arrow)

**Fig. 3:**
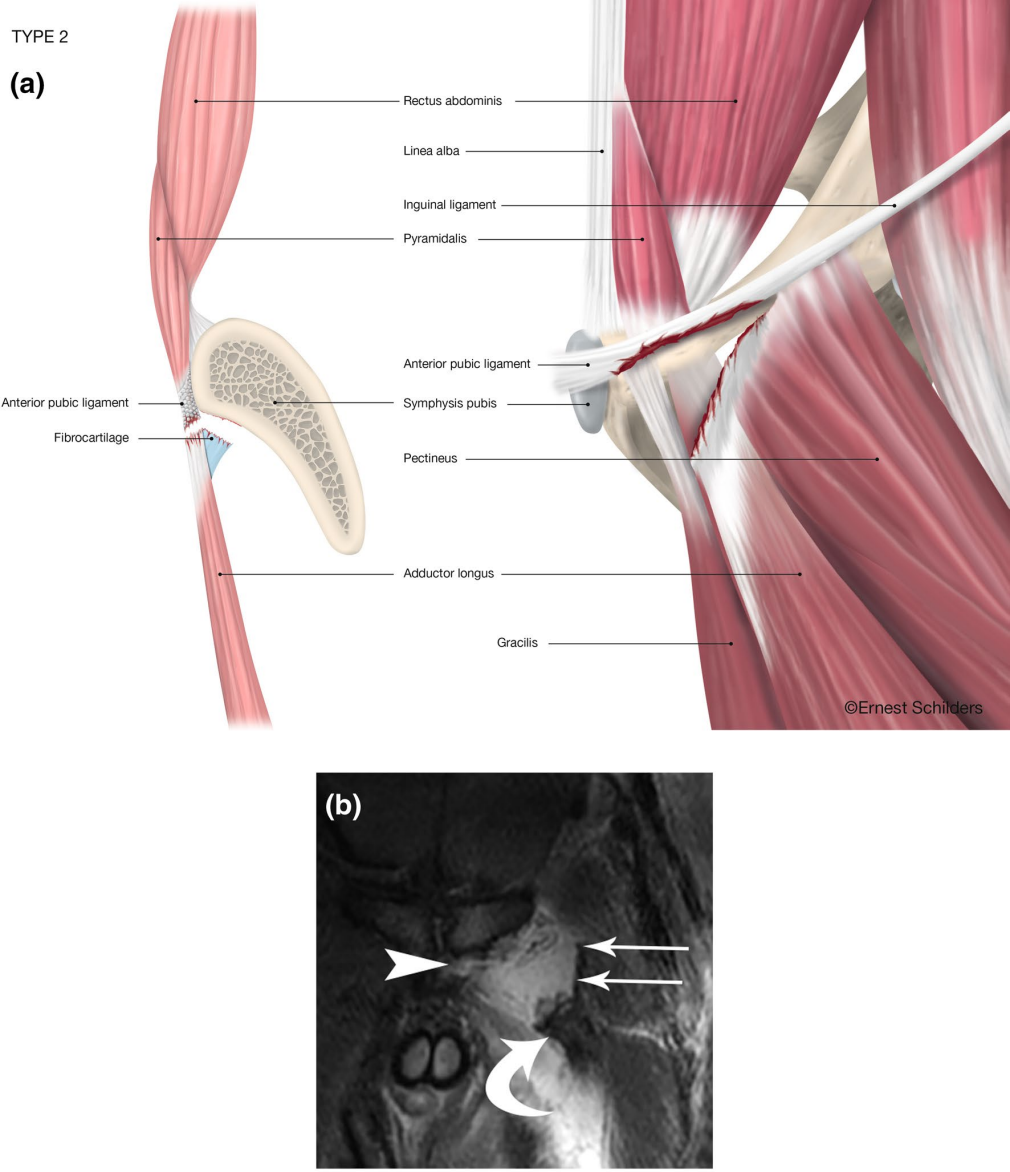
Type 2 PLAC injury: Complete FC avulsion–Pyramidalis separated from Adductor Longus–partial Pectineus tear. **a** Anatomical line drawing. **b** Coronal FST2 MRI. Adductor longus fibrocartilage torn and retracted laterally (curved arrow). Left side of anterior pubic ligament bridge torn (arrowhead). Pectineus muscle with a strip of inguinal ligament/lacunar ligament partially torn from pubic attachment and displaced laterally (straight arrows)

**Fig. 4:**
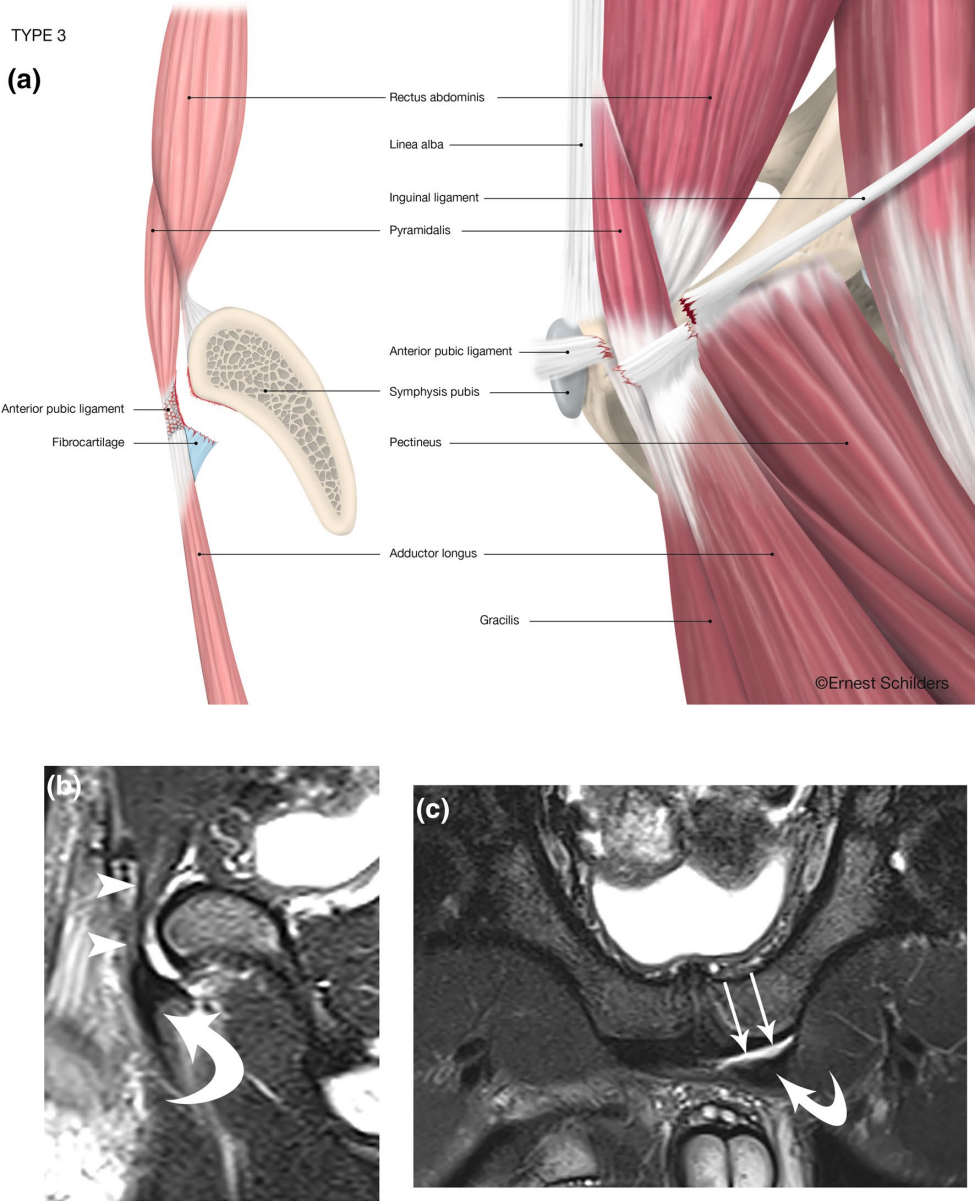
Type 3 PLAC injury: complete FC avulsion–Pyramidalis connected to Adductor Longus–intact Pectineus. **a** Anatomical line drawing. **b** Sagittal FST2 MRI. Pyramidalis is intact (arrowheads) and in continuity with anteriorly and inferiorly displaced adductor longus fibrocartilage (curved arrow). **c** Oblique axial FST2 MRI. The left adductor longus fibrocartilage is displaced anteriorly and laterally and separated by a fluid filled space (arrows) from the pubic attachment

**Fig. 5:**
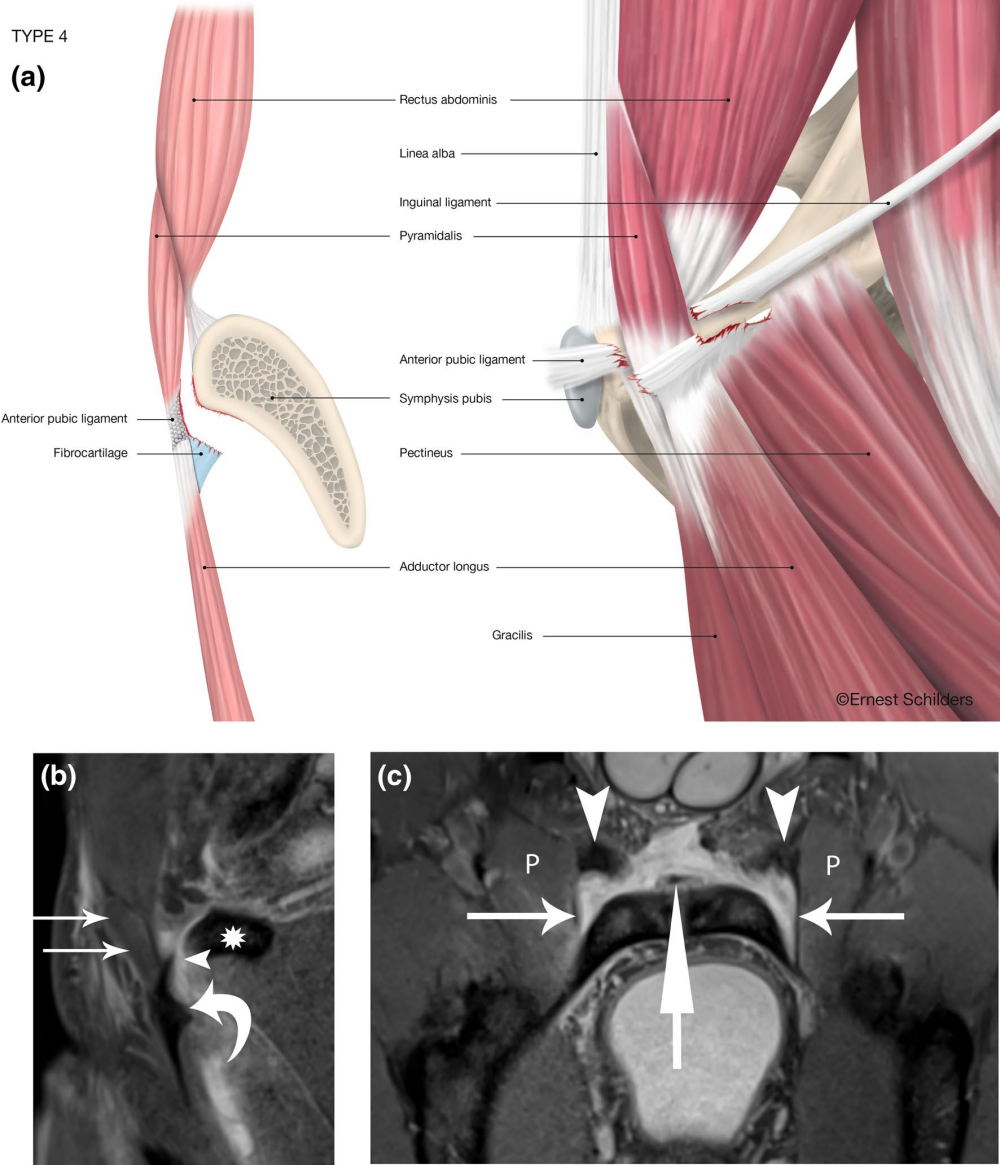
Type 4 PLAC injury: complete FC avulsion–Pyramidalis connected to Adductor Longus–partial Pectineus tear. **a** Anatomical line drawing. **b** Sagittal FSPD MRI. The pyramidalis muscle is in continuity with the adductor longus fibrocartilage (curved arrow) separated by a fluid space (arrowhead). The anterior pubic ligament is completely avulsed from the pubic bone (*). **c** Oblique axial FST2 MRI. Both adductor longus fibrocartilage are avulsed (arrowheads). Bilateral partial pectineus (P) tears are present with avulsion of the lacunar ligament (horizontal arrows). Centrally the internal tendon of the rectus abdominis (vertical arrow)

**Fig. 6:**
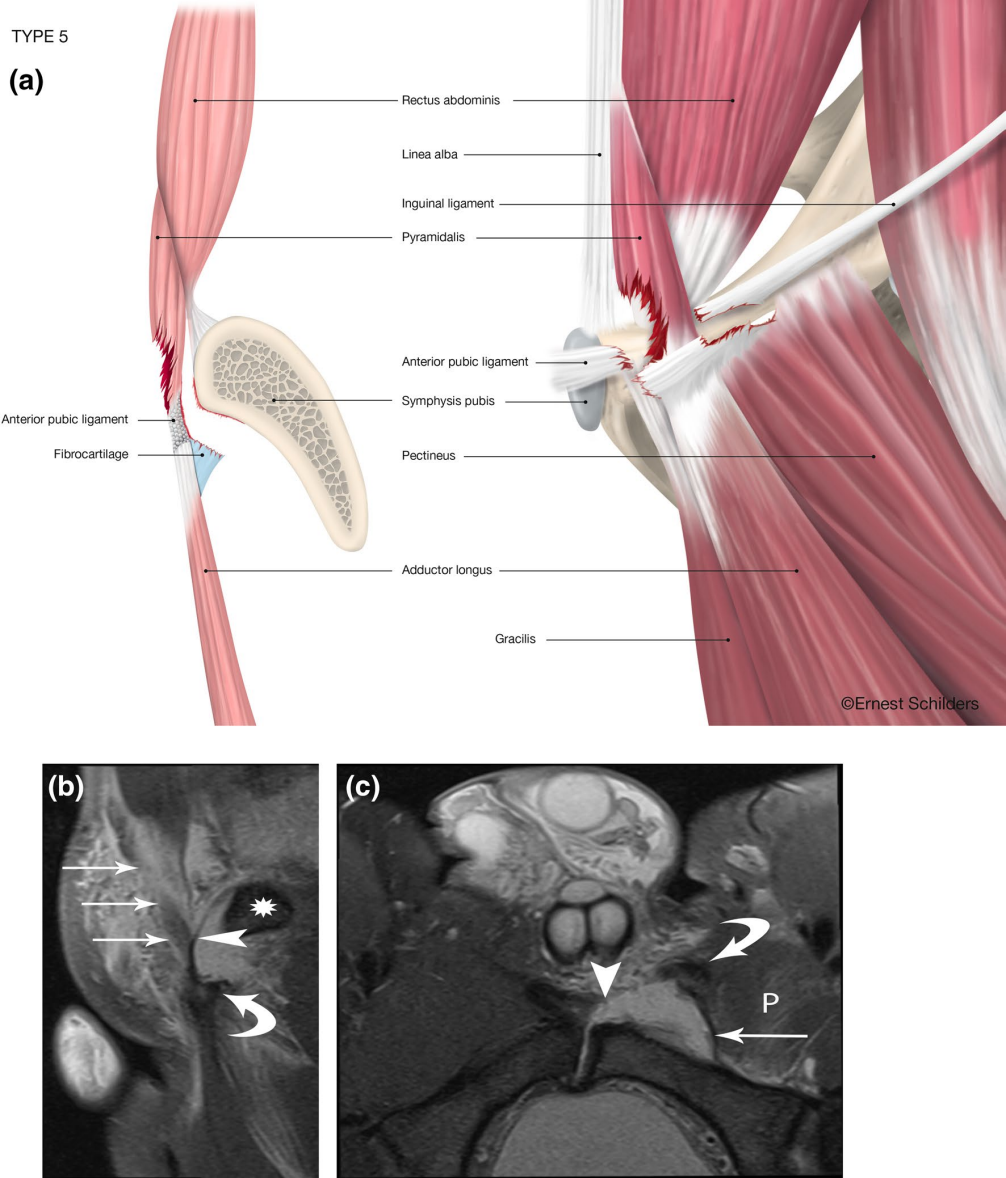
Type 5 PLAC injury: complete FC avulsion–Pyramidalis partially separated from Adductor Longus–partial Pectineus tear. **a** Anatomical line drawing. **b** Sagittal FSPD MRI. There is a partial tear through the pyramidalis muscle (arrows). Note the lacunar ligament (arrowhead) and superior pubic ramus (*). The adductor longus fibrocartilage is avulsed and displaced inferiorly (curved arrow). **c** Oblique axial FSPD MRI. The left fibrocartilage is avulsed anteriorly (curved arrow) and the left pectineus muscle (P) and lacunar ligament is partially torn from the anterior margin of the pubis (arrow). There is a tear through the anterior pubic ligament bridge over the symphyseal joint on left side (arrowhead)

**Fig. 7:**
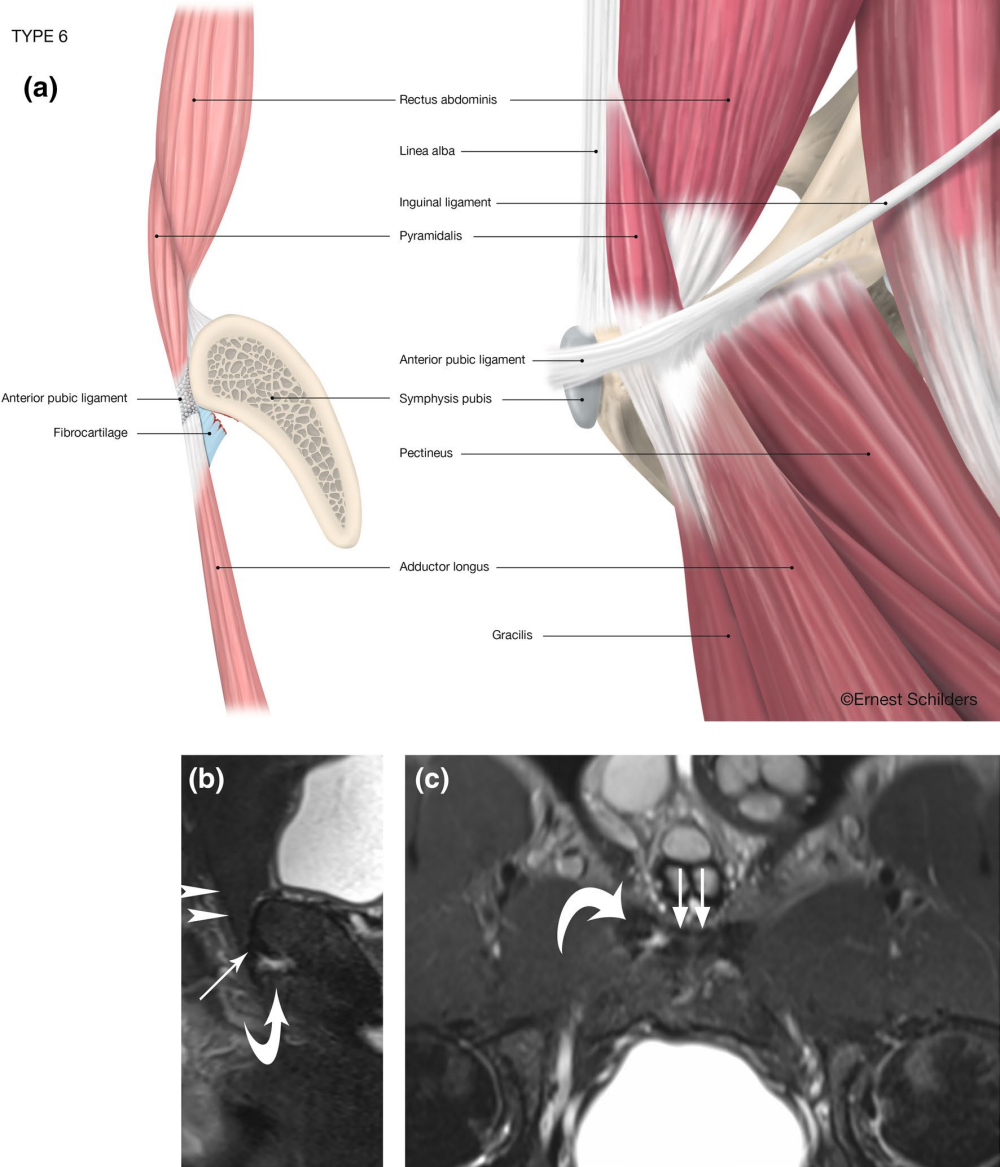
Type 6 PLAC injury: partial FC avulsion–Pyramidalis connected to Adductor Longus–intact Pectineus. **a** Anatomical line drawing. **b** Sagittal FST2 MRI. The pyramidalis (arrowheads) and anterior pubic ligament (arrow) are intact and in continuity with the adductor longus fibrocartilage (curved arrow). **c** Oblique axial FST2 MRI. There is a partial tear of the right adductor longus fibrocartilage (curved arrow). The anterior pubic ligament is intact (arrows)

Type 1 was used as the reference group on regressions because it has the largest number of patients. Table 3 demonstrates the association of disruption of the anterior pubic ligament (APL) crossing the symphyseal joint with the different types. Figures 2, 3, 4, 5, 6 and 7 illustrate the MRI imaging findings in each group.

**Table 3:** The incidence of anterior pubic ligament disruption varied between each subtype with high rates in types 2 and 4 and low rates in types 1 and 6

	No symphyseal APL disruption	Symphyseal APL disruption
Type 1	34 (70.8%)	14 (29.2%)
Type 2	17 (51.5%)	16 (48.5%)
Type 3	21 (60.0%)	14 (40.0%)
Type 4	3 (42.8%)	4 (57.2%)
Type 5	3 (50%)	3 (50%)
Type 6	12 (92.3%)	1 (7.7%)

The incidence of disruption of the anterior pubic ligament (APL) varied with each subtype (Table 3).

There was significant heterogeneity in the odds ratios (ORs) for APL by group (*p* = 0.05), with Type 6 less likely to have APL (OR = 0.20, 95% CI [0.01, 1.18]) and Type 2 more likely to have APL (OR = 2.29, 95% CI [0.91, 5.85]) than Type 1.

Similarly, the degree of displacement of the adductor longus tendon varies according to subtype. Minimal displacement was defined as less than 5 mm in any direction from the pubic attachment (Table 4). Type 3 PLAC lesion was associated with lower odds of adductor longus displacement (displacement vs minimal/none) (OR = 0.02, 95% CI [0.00, 0.07], *p* < 0.0001.

**Table 4:** The presence, absence and degree of adductor longus displacement vary between each PLAC injury subtype

	Minimal displacement	Displaced	No displacement
Type 1	4 (8.5%)	43 (91.5%)	0
Type 2	0	33 (100%)	0
Type 3	17 (48.6%)	6 (17.1%)	12 (34.3%)
Type 4	2 (28.6%)	5 (71.4%)	0
Type 5	0	6 (100%)	0
Type 6	1 (7.70%)	0	12 (92.3%)

The rectus abdominis was injured in 5 patients (3.5%). This only occurred in patients with a partial pectineus avulsion. In type 4 lesions, a rectus abdominis injury occurred in 42.8% of the cases.

There was no significant association with age at injury.

## Discussion

The most important finding of the present study was that imaging demonstrates that proximal adductor avulsion is rarely isolated but usually involves injury to the PLAC and pectineus.

The symphyseal anatomy has recently been redefined and the term PLAC is now recognised as the true anatomical relationship of the symphyseal groin anatomy [20]. Moreover, previous work has demonstrated that the various component parts of the PLAC and its related structures are well defined on MR imaging assuming the correct protocol has been followed [4]. This study allows us to use this new anatomical–radiological knowledge to help identify the injury patterns sustained in elite athletes, which subsequently has a significant impact on surgical planning and decision-making. This is the largest study of proximal adductor avulsion injuries in the literature, reporting on 145 cases. It is clear that in the majority of cases, adductor avulsions are not isolated injuries but injuries to the PLAC, often associated with partial pectineus tears. This implies a more significant injury and potentially more complex surgery to address all the injured components. This is the first study to highlight the concurrent involvement of the pyramidalis on MRI in adductor longus avulsions. A previous study by Serner et al. involving 16 cases of proximal avulsions reported that 25% of the cases were partial avulsions [25]. In our study of a larger cohort of patients only 9% of the avulsions were partial, and this referred specifically to the partial avulsion of the adductor longus fibrocartilage. We found that 34% of the athletes in our study had type 1 adductor longus fibrocartilage avulsion. Within this group 52% had increased T2 or STIR signal in the pyramidalis indicating a concurrent pyramidalis muscle injury. These findings contrast the earlier study [25] and provide the evidence within a larger number of patients that adductor longus avulsions are often associated with pyramidalis and/or partial pectineus avulsions.

The anterior pubic ligament bridges the symphyseal joint. There was a higher percentage of anterior pubic ligament injuries spanning the symphyseal joint when there was a partial pectineus avulsion. There is increased displacement of the adductor longus fibrocartilage when the adductor longus is partially or completely separated from the pyramidalis muscle. This displacement was even greater when there was an associated pectineus injury. The status of the pyramidalis muscle is, thus, shown to influence the degree of adductor longus displacement following an avulsion. An associated pectineus injury indicates a more significant injury.

The inguinal ligament is also known as Poupart’s ligament. At its medial attachment, there is a crescent shaped extension of fibres attaching into the pectineal line known as lacunar ligament also known as Gimbernat’s ligament [7, 8]. The pectineus muscle originates from the lacunar ligament and the linea ileo-pectinea. Anatomically the pectineus lies in the same plane as the adductor longus, and the muscles are typically blended together at their superior margin [7]. This explains the potential for an associated pectineus injury in the context of an adductor longus avulsion.

On MRI, the presence of longitudinal splitting of the inguinal ligament represents the separation of lacunar ligament, and is, therefore, representative of a partial avulsion of the pectineus.

The rectus abdominis muscle was rarely involved, only in 3.5% of the cases. The site of injury was always at the linea alba, and typically associated with displacement of the pyramidalis. The role of the rectus abdominis in relation to the adductor longus avulsions has previously been over reported [13] due to the incorrect anatomical concept that the distal rectus abdominis is anterior to the pubis and forms an aponeurosis with the adductor longus [12, 14, 29, 30]. Cadaveric studies have shown that the pyramidalis is the only abdominal muscle anterior to the pubis [18–20] and is commonly involved with PLAC injuries as demonstrated in this study.

Due to the anatomical complexity of the PLAC, the use of muscle classification systems such as the Munich consensus [11] does not fully determine the complexity or severity of the injury.

Equally, the DOHA classification [28] may not entirely define this injury. The principal abdominal muscle involved in PLAC injuries, the pyramidalis muscle, was not considered in this consensus. Patients often present with lower abdominal pain and bruising due to the pyramidalis involvement, thereby presenting with symptoms that overlap different anatomical regions to what is defined in the DOHA consensus.

There are significant variations in surgical techniques for the management of adductor avulsions. Defining the characteristics of PLAC injuries is a foundation for clearer anatomical and better surgical [5, 22] and non-surgical [21, 23] management of these injuries.

The findings of our study demonstrate that in patients with adductor avulsions, the other components of the PLAC should be assessed, including the connection with adductor longus. In addition, the pectineus and inguinal ligament should be routinely evaluated with these types of injuries. The correct magnetic resonance imaging protocol is essential to enable assessment of the PLAC. Radiologists need to be aware of the patterns of injury in addition to the recently described PLAC anatomy. Surgeons similarly need to be aware of these injury patterns when considering repair of these complex injuries.

Our group of patients has an overrepresentation of professional male athletes participating in football and rugby. The MR imaging characteristics of PLAC injuries in other sports may be different due to variation in mechanisms of injury as well as gender differences.

Currently the imaging review is not correlated with surgical findings; however, this is subject to a further study.

## Conclusion

The proximal adductor longus forms part of the PLAC and is rarely injured in isolation.

PLAC injury is a more appropriate term to describe this complex injury. An understanding of the anatomy and associated findings is key in the diagnosis of these often complex injuries. Radiologists and surgeons should be aware of the patterns of injury in relation to the recently described PLAC anatomy. This study will improve the MRI reporting and assist with the recognition of the complex patterns of PLAC injuries. The study will also enable the operating surgeon to accurately identify the various structures that need to be repaired.
